# Intervention components, mediators, and mechanisms of change of Internet- and mobile-based interventions for post-traumatic stress disorder: protocol for a systematic review and meta-analysis

**DOI:** 10.1186/s13643-019-1190-6

**Published:** 2019-11-07

**Authors:** Lena Steubl, Cedric Sachser, Harald Baumeister, Matthias Domhardt

**Affiliations:** 1 Department of Clinical Psychology and Psychotherapy, University of Ulm, Albert-Einstein-Allee-47, 89081 Ulm, Germany; 2 Clinic for Child and Adolescent Psychiatry/Psychotherapy, Ulm University Medical Center, Ulm, Germany

**Keywords:** Internet- and mobile-based, PTSD, Mediators, Intervention components, Mechanism of change

## Abstract

**Background:**

While Internet- and mobile-based interventions (IMIs) might possess the potential to increase access to evidence-based therapies for post-traumatic stress disorder (PTSD), comprehensive knowledge on active intervention components and change mechanisms underlying their efficacy is largely pending so far. The proposed systematic review and meta-analysis will systematically review the current status of research on the efficacy of IMIs for adult PTSD compared to active control conditions and identify active intervention components and mediators responsible for therapeutic change.

**Methods:**

A systematic literature search (PsycINFO, Medline/PubMed, Embase, CENTRAL, ICTRP, and Web of Science) will be conducted using keywords targeting “PTSD” and “Internet- and mobile-based interventions”. Two independent researchers will retrieve studies eligible for inclusion and extract and evaluate data (design, population, outcomes, sample size, duration of intervention and follow-up, drop-out rate). Risk of bias will be assessed, and results will be synthesized qualitatively and evaluated meta-analytically when possible.

**Discussion:**

The results of this systematic review and meta-analysis might further contribute to the development of IMIs for PTSD by highlighting intervention components and mediators associated with their efficacy. Knowledge about the active ingredients might ultimately lead to more effective interventions and treatment packages, with implications for clinical practice and dissemination of these rather novel interventions.

**Systematic review registration:**

PROSPERO (CRD42019130314).

## Background

Post-traumatic stress disorder (PTSD) is a burdensome [1] and highly prevalent disorder with an estimated 6.8% lifetime prevalence rate of PTSD for the adult US population [2]. PTSD also ranks under the 15 mental and neurological conditions that are most threatening for disability-adjusted life years in Europe [3], but prevalence rates and exposure to potential traumatic events may vary between countries [4]. In DSM-5, PTSD is classified as among the group of trauma- and stressor-related disorders following exposure to a traumatic or stressful event with the diagnostic criteria including at least one stressor (e.g., exposure to threatened death, sexual violence), intrusion symptoms, avoidance, negative alterations in cognitions and mood, alterations in arousal and reactivity, a duration of at least 1 month, functional significance, and exclusion of other explanatory factors (e.g., substance use [5]). The current draft of the ICD-11 diagnostic criteria suggests a symptom cluster of re-experiencing, avoidance, and enduring perceptions of heightened current threat; furthermore, the upcoming ICD-11 will also allow the diagnosis of complex PTSD which is additionally characterized by problems in affect regulation, beliefs about oneself as diminished, defeated, or worthless, accompanied by feelings of shame, guilt, or failure related to the traumatic event and difficulties in sustaining relationships and in feeling close to others [6].

Psychological interventions to treat PTSD are numerous with exposure-based therapy, cognitive processing therapy (CPT), cognitive therapy (CT), therapies based on cognitive behavioral therapy (CBT) principles, eye movement desensitization and reprocessing (EMDR), and narrative exposure therapy showing at least moderate effects ranging from standardized mean differences (SMD) of − 2.0 to − 0.3 [7]. Patient preferences demonstrate a threefold higher preference for psychological treatments compared to medication [8]. Still, although there is a well-established evidence-base for effective psychotherapeutic face-to-face treatments for this condition [7, 9], only a fractional number of patients receive treatment [10–12]. For example, in a survey interviewing US veterans with self-reported mental disorders (i.e., PTSD, generalized anxiety, major depression) after duty in Iraq and Afghanistan, only 23 to 40% stated that they received any professional help [10]. Another study examining asylum seekers in the Netherlands reports that 54% of the participants originating from Asian and African countries diagnosed with PTSD did not receive any treatment [13]. Reasons for the limited uptake of evidence-based treatments may be associated with fear of stigmatization or logistic barriers to treatment [10, 12]. Additionally, among those who actually do seek treatment, many do not receive evidence-based treatments or an adequate treatment dose [10].

Compared to traditional face-to-face intervention settings, using the Internet as a medium for the delivery of psychological interventions offers several advantages that might help to overcome treatment barriers [14]. Several meta-analyses showed their efficacy both for other mental disorders (e.g., depression or anxiety [15–19]) and PTSD in particular [20–22]. In addition, Internet- and mobile-based interventions (IMIs) are easy to access and offer users the opportunity to flexibly integrate the treatment into daily life, regardless of any limits in terms of space and time [23]. Moreover, users can set their own pace going through each session (potentially with automated feedback on their personal progress) as frequently or as quickly as they like [24]. Looking at PTSD in particular, online interventions can offer a unique and geographically independent treatment opportunity in underserved post-conflict areas [25]. It is also proposed that the anonymity in conduct of an online intervention for PTSD is one of its main advantages, particularly in some cultures (e.g., Arab cultures [25]). Correspondingly, one study has shown that anonymity, privacy, and confidentiality are the most frequently listed reasons for the use of the Internet for mental health support by survey respondents [26]. Additionally, studies also found IMIs on some mental health conditions such as depression to be scalable and cost-effective [14, 27, 28], a valid argument considering limited resources in many health care systems worldwide. Nevertheless, risks concerning IMIs (e.g., lacking adherence, non-detected negative outcomes, negative attitudes of both patients and clinicians towards these interventions, crisis management in case of anonymous users) should be carefully considered and comprehensively investigated [14].

However, given their presumed potential to overcome treatment gaps and advance mental health services on a large scale, a closer inspection of the evolving field of IMIs for PTSD is worthwhile. Gaining insight on the efficacy as well as on active intervention components (i.e., intervention components that are responsible for the efficacy and can therefore deemed active, e.g., human support, exposure, prompts, cognitive processing/restructuring) and mechanisms of change (i.e., processes or events responsible for the change of the outcome [29]) in IMIs may allow to further optimize outcomes and prevent possible adverse events [30–33].

To test their efficacy, IMIs can be compared with active or inactive controls. Considering the confounding situation in the latter designs with specific ingredients (i.e., variables (e.g., therapeutic techniques) unique to a certain form of psychotherapy [34]) becoming mixed up with common factors (i.e., active ingredients contributing to the outcome while being common to all forms of psychotherapy [34, 35]), this systematic review and meta-analysis focusses on active controls. For example, in the context of IMIs for PTSD, one study [36] compares a self-guided web-based CBT intervention for cancer patients with web-based information-only with results favoring the intervention. Moreover, these active controls can be classified as either bonafide (i.e., treatments that were intended to be therapeutic [37]) or non-bonafide. Hereby, it is particularly proposed to compare IMIs with common factor controls as active controls (i.e., structural equivalent packages that do not contain the specific components of the IMI hypothesized as the active verum of the intervention [38]).

In contrast, so-called component studies that compare one treatment with a treatment in which one specific component is left out (i.e., dismantling studies) or added (i.e., additive studies) offer the possibility to examine active intervention components and assess their incremental (add on) effect sizes [39]. For instance, there is a study [40] comparing a self-guided web-based intervention for war veterans with (subthreshold) PTSD and hazardous alcohol use with peer support with the same intervention without peer support, whereas no between-group differences are reported. Nevertheless, to date, there is ample evidence for guidance as a beneficial feature of Internet-based interventions [16, 31] with pending evidence for incremental effects of other single components [16].

Lastly, mechanisms of change (i.e., the actual processes responsible for therapeutic change) can be indicated by mediators (e.g., functional cognitions, emotional regulation, or problem-solving skills), and thus, the study of mediators is an important first step to understand mechanism of change [29]. Hence, a quantitative assessment of changes within a well-established mediation analysis is necessary in this context. For example, one study [41] assessed the mediating role of self-efficacy beliefs on PTSD symptom reduction in an Internet-based self-efficacy intervention and reports that health and human services professionals experiencing increased levels of self-efficacy are more likely to show lower secondary traumatic stress at follow-up. Another study on the mechanisms of change in CPT and prolonged exposure therapy for PTSD assessed habituation and hopelessness [42] while a study on mechanisms of change in IMIs irrespective of disorders concluded that cognitive factors are an important mechanism of change while assuming causal effects without mediation analyses [33]. This leaves room for an explorative assessment of intervention components and mediators and mechanisms of change specific for PTSD. In line with previous publications [29, 43, 44], we hereby conceptualize mediators as intervening variables that may statistically account for the relationship between the independent variable and the outcome. In contrast, moderators are variables or characteristics that influence the magnitude (or direction) of the relationship between an intervention and outcome [29], thereby indicating on whom and under which conditions treatments have differential effects [44]. As such, moderators suggest that different processes are involved (i.e., “moderated mediation”), but conceptually represent no mechanisms of therapeutic change themselves [29, 44].

Additionally, adherence is an important aspect aside symptom severity, considering both the high drop-out rates in IMIs for PTSD in various trials (e.g., 51.2% post-intervention [41]) and the hypothesized dose-response relationship for the use of IMIs (e.g., [45, 46]), which suggests an underestimation of effects in case of low adherence. Thus, reviewing adherence data in this field is highly valuable in order to gather knowledge on intervention components contributing to increased adherence rates and thereby inform intervention development.

To our knowledge, two meta-analyses in this field solely investigated telehealth interventions [47, 48], whereas three recent meta-analyses [20–22] investigated Internet-based interventions for posttraumatic stress and found evidence for their efficacy. In particular, one meta-analysis [20] found effects ranging from SMD = − .66 to − .83 while including 13 studies with passive and 7 with active comparison conditions and an overall sufficient quality of included studies reported by the authors. Another meta-analysis [21] compared Internet-based CBT (iCBT) to both waitlist/TAU controls (SMD = − .71; 10 studies) and other interventions (SMD = − .28; 3 studies), with authors reporting a varying methodological quality of included studies. A third meta-analysis [22] with 33 included studies reported a pooled SMD of − .35 favoring Internet-based interventions independent of the comparison condition while including 18 active and 20 waitlist/TAU comparisons and reporting a slightly increased effect size when studies with reported high risk of bias were excluded (8 comparisons). However, none of these meta-analyses distinguished between bonafide and non-bonafide active controls, and to the best of our knowledge, there has been no systematic review or meta-analysis on IMIs for PTSD examining intervention components in additive or dismantling studies. The existing meta-analyses only provide indirect evidence on a possible larger effect of IMIs with therapeutic support compared to those without therapeutic support [21] and no evidence for responsible intervention components [20, 22] while including (predominantly) passive comparison conditions. The same holds true for mediators or mechanisms of change that have not been meta-analytically summarized so far. Moreover, even though the results indicate that Internet-based treatments are effective, interventions designed as smartphone applications were excluded in former reviews [20, 21].

Thus, this review and meta-analysis will provide a broader and updated overview of this field of research, focusing on intervention components and mediators. The proposed systematic review and meta-analysis aims to systematically review, critically evaluate, and statistically integrate the existing literature on IMIs for PTSD in order to answer the following research questions:

1. Are IMIs for PTSD efficacious regarding symptom reduction?
  - Are IMIs for PTSD equally efficacious when compared to bonafide control conditions/interventions?
  - Are IMIs for PTSD more efficacious than attention/psychological placebos, or other non-bonafide active (online) control conditions?
2. Which intervention components are responsible for the efficacy of IMIs for PTSD?
3. What potential mediators and mechanisms of change in IMIs for PTSD are examined so far?

## Methods

This review and meta-analysis is registered with the International Prospective Register of Systematic Reviews (PROSPERO, CRD42019130314) and will be reported according to the Preferred Reporting Items for Systematic Reviews and Meta-Analyses (PRISMA) guidelines [41]. The PRISMA-P checklist can be found in Additional file 1.

### Search strategy

A systematic literature search in the electronic databases PsycINFO, Medline/PubMed, Embase, and CENTRAL (Cochrane Central Register of Controlled Trials) with no restriction on dates of coverage and language will identify relevant studies. The sensitive search strategy employs a combination of search terms including the target diagnosis PTSD and the type of treatment delivery (i.e., Internet- or mobile-based). The predefined set of search strings can be found in Appendix 1.

A review of reference lists from identified studies (i.e., backward searches), a search of the WHO International Clinical Trials Registry Platform (ICTRP; Appendix 2) for ongoing studies with preliminary/unpublished results, and a citation search using Web of Science will complement the search. If necessary, study authors will be contacted to obtain further information regarding study characteristics. In case study protocols are identified without subsequent publication of results, protocol authors will be contacted to obtain missing or unpublished data and determine eligibility for inclusion in this review and meta-analysis.

### Eligibility criteria

#### Population

Studies are eligible for inclusion if they focus on an adult target population (≥ 18 years; no upper limit) with PTSD or subthreshold PTSD assessed by standardized diagnostic interviews, observer-rated instruments with normed cutoff points, or validated self-reports (see Types of Outcomes). Studies of participants with different mental disorders (e.g., generalized anxiety disorder) will be included only if PTSD symptom severity is defined as primary outcome or main diagnosis of interest and results are reported specific to the respective disorders.

#### Intervention

Studies will be included if at least one trial arm constitutes of an Internet- and mobile-based intervention (IMI) targeting PTSD symptoms. IMIs are defined as self-guided/unguided or guided interventions delivered via Internet- and mobile-based communication technology with a psychological/psychotherapeutic focus (e.g., trauma-focused CBT, CPT-based interventions [49, 50]). Interventions may vary concerning to the amount of guidance provided to participants (i.e., pure self-help interventions as well as guided interventions with different amounts and types of human support will be included). The following variations of IMIs are all eligible for inclusion: (a) guided interventions (i.e., with regular therapist contact), (b) mostly unguided interventions (i.e., predominantly self-help, e.g., with additional technical guidance on demand), and (c) completely unguided interventions (i.e., self-help, with no therapeutic support but assessment at most [51]). Therefore, studies are eligible for inclusion if IMIs involved an initial face-to-face interview or an initial face-to-face session.

#### Comparison

Eligible comparisons differ by each research question. First, to investigate the efficacy of IMIs for PTSD, studies must include an active control group. Second, to investigate intervention components, studies must classify as additive or dismantling design study (see the “Study type” section). Third, to investigate mediators and mechanisms of change, studies are eligible for inclusion if they compare an IMI group with an active (e.g., IMI, face-to-face, treatment as usual, or placebo) or non-active control group (e.g., wait-list or no intervention).

To be classified as bonafide [37] in this systematic review and meta-analysis, the treatment for the control groups has to aim to reduce symptom severity (non-inferiority trials). If the authors postulate a superiority of the IMI group, we are classifying the control group as non-bonafide. In case authors do not specify their hypotheses clearly, we will decide the allocation depending on the existence of empirical evidence for the efficacy of the control treatment.

#### Outcome

##### Primary outcome

Change in PTSD symptom severity has to be measured by scores on a standardized, observer-rated instrument, for example, the Clinician-Administered PTSD Scale for DSM-IV (CAPS [52]), or a validated self-report measure of PTSD symptoms, for example, the Posttraumatic Stress Disorder Checklist - DSM-5 Version (PCL-5 [53]) or precursor version. Negative values characterize effect sizes favoring the intervention group.

##### Secondary outcomes

To depict the influence of the participants’ engagement with the online intervention, adherence will be operationalized as (a) the mean number of main intervention units completed (e.g., lectures, diaries) and (b) the percentage of participants that completed the whole treatment [54, 55].

##### Possible mediators

Possible mediators (e.g., functional cognitions, mindfulness, or problem-solving skills) are to be measured by validated psychometric instruments (e.g., Posttraumatic Cognitions Inventory [56]). Hereby, mediators are defined as intervening variables that statistically account for the relationship between an independent (i.e., the treatment) and a dependent variable (i.e., the outcome [29]).

##### Timing of outcome assessment

To be able to meaningfully comment on any post-intervention symptom reduction, separate analyses based on different periods of assessment will be performed (e.g., immediately post-treatment and after the follow-up period). The resulting follow-up periods will be clustered in three time frames: short-term (1–3 months after post-treatment), medium-term (> 3 to ≤ 12 months after post-treatment), and long-term (> 12 months after post-treatment) effects. If studies report more than one follow-up assessment point, the longest follow-up period will be used to provide the best estimate of the crucial long-term outcomes of the PTSD intervention.

#### Study type

In order to investigate the efficacy of IMIs for PTSD and active intervention components, only randomized controlled trials (RCTs [57]) with active control groups or comparing IMIs to dismantled variations of the same intervention will be included. The latter allows to investigate specific effects of single components of IMIs by adding or subtracting specific elements [58]. To study potential mediator variables, both original RCTs and secondary analyses on previous RCTs will be eligible. They have to include repeated measures and use well-established mediation analyses (e.g., [59]) or include a quantitative assessment of changes in investigated psychological mediators. Studies have to be written in English and either be published in peer-reviewed journals or classified as ongoing trials in ICTRP with already existing results.

### Study selection process

Two independent reviewers (LS, AS) will conduct the selection of articles. First, one reviewer (LS) will screen all titles and abstracts of articles yielded by the database search. Second, full texts of the selected articles will be retrieved and screened by both reviewers (LS, AS) in terms of the aforementioned eligibility criteria. Third, reference lists of finally included articles will be screened in the same way. Disagreement will be resolved by discussion among reviewers or, if necessary, by consultation of a third reviewer (MD). To illustrate the study selection process and reasons for exclusion, a PRISMA-P flow chart [60] will be provided. Records will be managed using the literature management program CITAVI 6.

### Data extraction

The following data items will be extracted by two independent reviewers (LS, AS) for each study: (a) study identification items (year of publication, first author, country), (b) study and intervention characteristics (inclusion and exclusion criteria, screening instruments, therapeutic background, sample size, intervention design/type, level of human support/guidance, control group, duration of intervention/number of sessions, follow-up assessments, recruitment strategy, communication mode, prompts, standardization), (c) target population items (gender, age, specific population groups if applicable, comorbidities, trauma type, type of PTSD), (d) setting (nationality, environment, recruitment strategy), (e) drop-out rate, (f) human support characteristics (e.g., qualification of e-coaches if any), (g) platform characteristics, (h) frequency of adverse events if assessed and reported in primary studies, and (i) dimensional clinical outcomes.

In case of overlapping studies or multiple studies on the same data set, all information will be extracted with a note, highlighting the shared data set. Should there be any data missing or the reported data unclear, study authors will be contacted and asked for further clarification.

In case of multiple outcome measures for the assessment of PTSD symptom severity, the primary outcome measure of the study will be selected. If outcomes are assessed by several instruments, data will be extracted as follows: (a) the primary outcome measure of the study will be prioritized; (b) in case of multiple outcome measures of the same hierarchical level, the most used outcome measure will be chosen for the meta-analysis; and (c) should the aforementioned steps be not be possible, we will randomly select one outcome measure.

### Quality assessment

Two independent reviewers (LS, AS) will assess the risk of bias using the Cochrane Collaboration’s tool for assessing risk of bias in RCTs [61] in order to evaluate the quality of included studies. As recommended, each study will be rated in the following domains: (a) random sequence generation, (b) allocation concealment, (c) blinding of participants and personnel, (d) blinding of outcome assessment, (e) incomplete outcome data, and (f) other bias (e.g., study has been claimed to be deceitful, baseline differences between intervention and control group). Studies will be rated as showing an “unclear”, “low”, or “high” risk of bias on each domain. Inter-rater reliability will be calculated by means of Cohen’s kappa, whereby a value between .60 and .80 can be considered as substantial and a value > .80 as perfect [62]. It is important to note that the third domain on blinding of participants and personnel is not warranted in (guided) IMIs, which would result in a high risk of bias rating. To prevent a distorted rating, we will rate this domain as “unclear” if this is the case.

To examine possible publication bias, the trim and fill procedure [63, 64], Egger’s test of funnel plot asymmetry [61], and visual inspection of funnel plots will be utilized.

Additionally, mediation studies will be qualitatively rated with the following criteria, originally proposed by Kazdin [29]: (a) underlying theory or conceptual model, (b) RCT and inclusion of a control group, (c) sufficient sample size per condition (defined as *n* ≥ 40), (d) examination of multiple mediators in one study, (e) assessment of temporality (defined as ≥ 3 assessments in treatment phase), and (f) direct experimental manipulation of the mediator.

### Data synthesis and presentation

Both text and tables will provide a detailed description of the results for all included studies. Characteristics of selected studies will be listed and qualitatively described. Characteristics include (a) study design and characteristics (sample size, duration, follow-up period) and patient population (age, gender, specific population group), (b) intervention characteristics (name, intervention content (e.g., CBT)), (c) technical implementation (e.g., Internet-only or Internet- and mobile-based), (d) duration, (e) level of human support/guidance, (f) study and intervention drop-out rate, (g) assessment tool used to determine presence of PTSD (clinical interview, questionnaire), (h) recruitment procedure, and (i) any covariates assessed (list of variables).

### Data analysis

To assess the efficacy of IMIs compared to active control conditions and the efficacy of their intervention components, data analyses will be performed using the Review Manager 5.3 software developed by the Cochrane Collaboration [65]. Meta-analytic pooling will be conducted when at least three studies report outcome parameters. If applicable, random-effects meta-analyses will be used to compute overall estimates of treatment outcomes. Standardized mean differences (SMD) and their 95% confidence intervals (CI) will be calculated for all continuous outcomes. As Hedges’ *g* is less biased than Cohen’s *d* in small samples [66], SMD will be given as values of the former measure. Dichotomous outcomes will be analyzed using risk ratios and corresponding 95% CIs and completer rate using odds ratios and their 95% CIs.

Heterogeneity will be evaluated with the *I*^2^ statistic, whereby an *I*^2^ value < 40% may not be important, a value between 30 and 60% may represent moderate heterogeneity, between 50 and 90% substantial heterogeneity, and > 75% considerable heterogeneity [57].

A forest plot will be created and used to visually investigate the presence and nature of statistical heterogeneity. In addition, statistical heterogeneity of the effect sizes will be evaluated using the *Q* statistic [67], with a significant *Q* indicating heterogeneity across studies that warrants further exploration.

For further comparisons of intervention and study characteristics (concerning, e.g., content, form, therapeutic background, guidance, trauma type, study quality, target population, and active intervention components), subgroup analysis will be performed if feasible. The possible influence of publication bias will be determined by inspection of funnel plots if feasible [57]. Feasibility will be given with at least three trials per subgroup for subgroup analysis and at least ten trials to determine possible influence of publication bias [57].

Sensitivity analyses will be conducted to test the robustness of the results by comparing the pooled SMD of the different times of outcome assessment (see the “Timing of outcome assessment” section): (1) short term, (2) medium term, and (3) long term. Further sensitivity analyses will be conducted to examine the effect of including studies at high risk of bias. Should a quantitative synthesis not be appropriate, results will be summarized qualitatively.

To evaluate and synthesize evidence for possible mediators, a two-stage structural equation modeling (TSSEM) approach employing R will be used if appropriate [68, 69]. This approach combines meta-analytic techniques and structural equation modeling (SEM) and has been considered superior to conventional approaches to synthesize studies that use SEM (e.g., Pearson correlations, generalized least squares [70]). Means, SD, *t* statistics, *F* statistics, and effect sizes will be used to calculate bivariate correlations if studies do not report bivariate correlation between treatment, mediator, and outcome [66]. Should a quantitative synthesis not be appropriate, results will be summarized qualitatively by summarizing the (significant) mediators found.

To measure the confidence in the cumulative evidence, the strength of the body of evidence will be assessed using the GRADE approach [71]. This system classifies the quality of evidence in four levels ranging from “very low” and “low” to “moderate” and “high” (influenced by, e.g., study limitations, inconsistency of results) and offers “strong” and “weak” as grades of recommendation (influenced by, e.g., quality of evidence, uncertainty, or variability in values and preferences [71]).

## Discussion

This systematic review and meta-analysis will add to previous research by summarizing, synthesizing, and discussing the existing literature on Internet- and mobile-based interventions for PTSD. The findings of this review will go beyond previous meta-analyses and systematic reviews in this field (e.g., [20–22]), as results will extend to intervention components and mediators and mechanisms of change, while also covering mobile-based interventions. By identifying and quantifying the active intervention components and mediators and mechanisms of change, it may advance our understanding on how Internet- and mobile-based intervention take effect. This can help to develop more effective interventions and prevent possible adverse events. Moreover, results might assist in the verification and advancement of psychotherapeutic theories and the development of an extensive model on how IMIs for PTSD and psychotherapies in general might work [29, 72].

However, there are several limitations to the proposed systematic review and meta-analysis. One important limitation might be caused by substantial heterogeneity of included studies. The clinical, methodological, and statistical differences might limit the possibility of quantitatively pooling trials as well as the generalizability of findings. Previous meta-analyses on IMIs for PTSD have shown up to considerably high heterogeneity while suggesting differences in employed therapeutic techniques among others to be responsible (e.g., [22]). Moreover, only studies written in English will be included, which may lead to an overestimation of effects as trials with statistically significant results have been shown to be more likely published in English [73]. Furthermore, despite the attempts to include unpublished and non-significant studies, the proposed systematic review and meta-analysis might also be limited by publication bias.

In sum, given the substantial burden of disease associated with PTSD and the opportunities of IMIs for extending and augmenting available service supplies, the proposed review and meta-analysis is urgently needed and will substantially add to the current evidence. The results will inform intervention development by highlighting active intervention components and mediators responsible for therapeutic change, and extend the evidence base on the efficacy of IMIs for PTSD.

### Supplementary information

**Additional file 1.** PRISMA-P 2015 Checklist. This checklist has been adapted for use with systematic review protocol submissions to BioMed Central journals from Table 3 in Moher D et al.: Preferred reporting items for systematic review and meta-analysis protocols (PRISMA-P) 2015 statement. Systematic Reviews 2015 4:1. An Editorial from the Editors-in-Chief of Systematic Reviews details why this checklist was adapted - Moher D, Stewart L & Shekelle P: Implementing PRISMA-P: recommendations for prospective authors. Systematic Reviews 2016 5:15.

## Data Availability

The datasets used and/or analyzed during the current study are available from the corresponding author on reasonable request.

## Appendix 1

**Table 1:** Search strings for PsycINFO/PSYNDEX, Medline, Embase, and CENTRAL (Ovid)

	PsycINFO/PSYNDEX	Medline	Embase	CENTRAL
S1	exp Posttraumatic Stress Disorder/	exp Stress Disorders, Traumatic/	exp posttraumatic stress disorder	exp Stress Disorders, Post-Traumatic/
S2	-	-	Psychotrauma/	-
S3	stress adj3 disorder.ab,ti,id.	stress adj3 disorder.ab,ti,kw.	stress adj3 disorder.ab,ti,kw.	stress adj3 disorder.ab,ti,kw.
S4	traumatic adj3 stress.ab,ti,id.	traumatic adj3 stress.ab,ti,kw.	traumatic adj3 stress.ab,ti,kw.	traumatic adj3 stress.ab,ti,kw.
S5	ptsd.ab,ti,id.	ptsd.ab,ti,kw.	ptsd.ab,ti,kw.	ptsd.ab,ti,kw.
S6	1 or 3 or 4 or 5	1 or 3 or 4 or 5	1 or 2 or 3 or 4 or 5	1 or 3 or 4 or 5
S7	exp online therapy/	-	-	-
S8	internet*.ab,ti,id.	internet*.ab,ti,kw.	internet*.ab,ti,kw.	internet*.ab,ti,kw.
S9	online*.ab,ti,id.	online*.ab,ti,kw.	online*.ab,ti,kw.	online*.ab,ti,kw.
S10	web*.ab,ti,id.	web*.ab,ti,kw.	web*.ab,ti,kw.	web*.ab,ti,kw.
S11	digital*.ab,ti,id.	digital*.ab,ti,kw.	digital*.ab,ti,kw.	digital*.ab,ti,kw.
S12	virtual*.ab,ti,id.	virtual*.ab,ti,kw.	virtual*.ab,ti,kw.	virtual*.ab,ti,kw.
S13	computer*.ab,ti,id.	computer*.ab,ti,kw.	computer*.ab,ti,kw.	computer*.ab,ti,kw.
S14	mobile*.ab,ti,id.	mobile*.ab,ti,kw.	mobile*.ab,ti,kw.	mobile*.ab,ti,kw.
S15	smartphone*.ab,ti,id.	smartphone*.ab,ti,kw.	smartphone*.ab,ti,kw.	smartphone*.ab,ti,kw.
S16	email*.ab,ti,id.	email*.ab,ti,kw.	email*.ab,ti,kw.	email*.ab,ti,kw.
S17	e-mail*.ab,ti,id.	e-mail*.ab,ti,kw.	e-mail*.ab,ti,kw.	e-mail*.ab,ti,kw.
S18	tele*.ab,ti,id.	tele*.ab,ti,kw.	tele*.ab,ti,kw.	tele*.ab,ti,kw.
S19	app.ab,ti,id.	app.ab,ti,kw.	app.ab,ti,kw.	app.ab,ti,kw.
S20	cyber.ab,ti,id.	cyber.ab,ti,kw.	cyber.ab,ti,kw.	cyber.ab,ti,kw.
S21	exp Telemedicine/	exp Telemedicine/	exp telehealth/	exp telemedicine/
S22	exp computer assisted therapy/	exp therapy, computer-assisted/	exp computer assisted therapy/	exp Therapy, Computer-Assisted/
S23	7 or 8 or 9 or 10 or 11 or 12 or 13 or 14 or 15 or 16 or 17 or 18 or 19 or 20 or 21 or 22	8 or 9 or 10 or 11 or 12 or 13 or 14 or 15 or 16 or 17 or 18 or 19 or 20 or 21 or 22	8 or 9 or 10 or 11 or 12 or 13 or 14 or 15 or 16 or 17 or 18 or 19 or 20 or 21 or 22	8 or 9 or 10 or 11 or 12 or 13 or 14 or 15 or 16 or 17 or 18 or 19 or 20 or 21 or 22
S24	psychotherap*.ab,ti.	psychotherap*.ab,ti.	psychotherap*.ab,ti.	psychotherap*.ab,ti.
S25	therap*.ab,ti.	therap*.ab,ti.	therap*.ab,ti.	therap*.ab,ti.
S26	treat*.ab,ti.	treat*.ab,ti.	treat*.ab,ti.	treat*.ab,ti.
S27	intervention*.ab,ti.	intervention*.ab,ti.	intervention*.ab,ti.	intervention*.ab,ti.
S28	self-help.ab,ti.	self-help.ab,ti.	self-help.ab,ti.	self-help.ab,ti.
S29	narrative exposure.ab,ti.	narrative exposure.ab,ti.	narrative exposure.ab,ti.	narrative exposure.ab,ti.
S30	EMDR.ab,ti.	EMDR.ab,ti.	EMDR.ab,ti.	EMDR.ab,ti.
S31	(eye movement desensitization and reprocessing).ab,ti.	(eye movement desensitization and reprocessing).ab,ti.	(eye movement desensitization and reprocessing).ab,ti.	(eye movement desensitization and reprocessing).ab,ti.
S32	CBT.ab,ti.	CBT.ab,ti.	CBT.ab,ti.	CBT.ab,ti.
S33	psychodynamic*.ab,ti.	psychodynamic*.ab,ti.	psychodynamic*.ab,ti.	psychodynamic*.ab,ti.
S34	(behav* adj2 activation*).ab,ti.	(behav* adj2 activation*).ab,ti.	(behav* adj2 activation*).ab,ti.	behav* activation*.ab,ti.
S35	ACT.ab,ti.	ACT.ab,ti.	ACT.ab,ti.	ACT.ab,ti.
S36	IPT.ab,ti,id.	IPT.ab,ti,kw.	IPT.ab,ti,kw.	IPT.ab,ti,kw.
S37	social skill* training.ab,ti.	social skill* training.ab,ti.	social skill* training.ab,ti.	social skill* training.ab,ti.
S38	(physical adj2 activit*).ab,ti.	(physical adj2 activit*).ab,ti.	(physical adj2 activit*).ab,ti.	physical activit*.ab,ti.
S39	(cognitive adj3 modification*).ab,ti.	(cognitive adj3 modification*).ab,ti.	(cognitive adj3 modification*).ab,ti.	cognitive modification*.ab,ti.
S40	24 or 25 or 26 or 27 or 28 or 29 or 30 or 31 or 32 or 33 or 34 or 35 or 36 or 37 or 38 or 39	24 or 25 or 26 or 27 or 28 or 29 or 30 or 31 or 32 or 33 or 34 or 35 or 36 or 37 or 38 or 39	24 or 25 or 26 or 27 or 28 or 29 or 30 or 31 or 32 or 33 or 34 or 35 or 36 or 37 or 38 or 39	24 or 25 or 26 or 27 or 28 or 29 or 30 or 31 or 32 or 33 or 34 or 35 or 36 or 37 or 38 or 39
S41	ICBT.ab,ti,id.	ICBT.ab,ti,kw.	ICBT.ab,ti,kw.	ICBT.ab,ti,kw.
S42	CCBT.ab,ti,id.	CCBT.ab,ti,kw.	CCBT.ab,ti,kw.	CCBT.ab,ti,kw.
S43	e-therap*.ab,ti,id.	e-therap*.ab,ti,kw.	e-therap*.ab,ti,kw.	e-therap*.ab,ti,kw.
S44	etherap*.ab,ti,id.	etherap*.ab,ti,kw.	etherap*.ab,ti,kw.	etherap*.ab,ti,kw.
S45	ecological momentary intervention*.ab,ti,id.	ecological momentary intervention*.ab,ti,kw.	ecological momentary intervention*.ab,ti,kw.	ecological momentary intervention*.ab,ti,kw.
S46	41 or 42 or 43 or 44 or 45	41 or 42 or 43 or 44 or 45	41 or 42 or 43 or 44 or 45	41 or 42 or 43 or 44 or 45
S47	(23 and 40) or 46	(23 and 40) or 46	(23 and 40) or 46	(23 and 40) or 46
S48	6 and 47	6 and 47	6 and 47	6 and 47
	exp = Explode -> "retrieve results using the selected term and all of its more specific terms"	exp = Explode -> "retrieve results using the selected term and all of its more specific terms"	exp = Explode -> "retrieve results using the selected term and all of its more specific terms"	exp = Explode -> "retrieve results using the selected term and all of its more specific terms"
	ti = Title	ti = Title	ti = Title	ti = Title
	ab = Abstract	ab = Abstract	ab = Abstract	ab = Abstract
	id = Key Concepts	kw = Keyword	kw = Keyword	kw = Keyword
	pt = Publication Type	pt = Publication Type	pt = Publication Type	pt = Publication Type
	md = Methodology			
	adj3 = adjacent within 3 words	adj3 = adjacent within 3 words	adj3 = adjacent within 3 words	

## Appendix 2

**Table 2:** Search strings for ICRTP

	Advanced search
S1	Title = (internet OR online OR web OR virtual OR email OR computer OR mobile OR smartphone)
S2	Condition = (PTSD OR stress disorder OR traumatic stress OR post-traumatic stress disorder OR post traumatic stress disorder)
S3	Intervention = (psychotherapy OR treatment)
S4	1 and 2 and 3

## Abbreviations

CAPS: Clinician-Administered PTSD Scale
CBT: Cognitive behavioral therapy
CENTRAL: Cochrane Central Register of Controlled Trials
CPT: Cognitive processing therapy
CT: Cognitive therapy
DSM: Diagnostic and Statistical Manual of Mental Disorders
EMDR: Eye movement desensitization and reprocessing
GRADE: Grading of Recommendations Assessment, Development and Evaluation
iCBT: Internet-based CBT
ICD: International Statistical Classification of Diseases and Related Health Problems
ICTRP: International Clinical Trials Registry Platform
IMIs: Internet- and mobile-based interventions
PCL-5: Posttraumatic Stress Disorder Checklist—DSM-5 Version
PRISMA: Preferred Reporting Items for Systematic Reviews and Meta-Analyses
PTSD: Post-traumatic stress disorder
RCT: Randomized controlled trial
SMD: Standardized mean difference
TSSEM: Two-stage structural equation modeling
